# Comparison of Molecular and In Silico *Salmonella* Serotyping for *Salmonella* Surveillance

**DOI:** 10.3390/microorganisms9050955

**Published:** 2021-04-29

**Authors:** Linda Chui, Christina Ferrato, Vincent Li, Sara Christianson

**Affiliations:** 1 Department of Laboratory Medicine and Pathology, University of Alberta, Edmonton, AB T6G 2R3, Canada; 2 Alberta Precision Laboratories-Public Health Laboratory (ProvLab), Edmonton, AB T6G 2J2, Canada; vincent.li@albertaprecisionlabs.ca; 3 Alberta Precision Laboratories-Public Health Laboratory (ProvLab), Calgary, AB T2N 4W4, Canada; christina.ferrato@albertaprecisionlabs.ca; 4 National Microbiology Laboratory, Public Health Agency of Canada, Winnipeg, MB R3E 3M4, Canada; sara.christianson@canada.ca

**Keywords:** *Salmonella* surveillance, serotyping, microarray, whole genome sequencing, public health

## Abstract

*Salmonella* surveillance and outbreak management is a key function of public health. Laboratories are shifting from antigenic serotype determination to molecular methods including microarray or whole genome sequencing technologies. The objective of this study was to compare the Check&Trace Salmonella™ DNA microarray (CTS), a commercially available assay with the *Salmonella* in silico typing resource (SISTR), which uses whole genome sequencing technology for serotyping clinical *Salmonella* strains in Alberta, Canada, collected over an 18-month period. A high proportion of isolates (96.3%) were successfully typed by both systems. SISTR is a powerful tool for laboratories which already have a WGS infrastructure in place, whereas smaller laboratories can benefit from a commercial microarray system and reduce the processing cost per isolate compared to traditional serotyping.

## 1. Introduction

*Salmonella* is a foodborne pathogen and a major source of gastrointestinal infection, with an estimated 93.8 million cases per year [1]. Salmonellosis remains a significant cause of morbidity and mortality, particularly in susceptible populations such as immunocompromised individuals, the elderly, and infants and young children. Public health surveillance reduces the public health burden of salmonellosis by rapidly detecting outbreaks, identifying sources, limiting transmission, and preventing future occurrences. In Canada, PulseNet is a vital surveillance system for pathogens associated with foodborne disease. The Alberta Precision Laboratories—Public Health Laboratory (ProvLab) in Alberta is a PulseNet Canada member that plays an important role in supporting PulseNet surveillance initiatives and outbreak management by serotyping human clinical *Salmonella* isolates submitted by laboratories across the province. Serotyping is crucial for the rapid and accurate detection of *Salmonella* outbreaks and is also integral to the resolution process. Historically, *Salmonella* serotyping was performed using the White–Kauffman–Le Minor (WKL) scheme, which is based on immunological reactions to somatic (O) and flagellar (H) antigens [2]. Although useful, recent technological advances in laboratory diagnostics have produced viable alternatives to conventional serotyping—a test that can be time-consuming, costly, and can produce results that are susceptible to subjective interpretation.

The Check&Trace *Salmonella*™ (CTS) assay (Check-Points, Wageningen, Netherlands) is a commercial DNA microarray system for *Salmonella* serotyping and is a reliable alternative to conventional methods [3]. The CTS system is based on the targeted amplification and detection of genetic markers followed by hybridization and automated comparison to an established database for serotype identification [4]. Serotyping using CTS is rapid and easy to implement in frontline microbiology as well as public health laboratories, and is comparably accurate relative to traditional methods. From 1 March 2015 to 3 February 2020, CTS was the primary method for *Salmonella* serotyping at the ProvLab.

An increasing number of public health laboratories worldwide have transitioned to next generation sequencing (NGS) for pathogen surveillance [5]. NGS data can be used to assess genetic similarity and evaluate genetic characteristics, such as virulence and antimicrobial resistance [6]. Further, NGS is capable of providing a previously unprecedented level of discrimination between genetically related isolates, and allows for extensive phylogenetic analysis [7,8]. These features are particularly useful for *Salmonella* serotypes that have a high degree of genetic similarity (i.e., *Salmonella* Enteritidis) and are difficult to differentiate using traditional molecular typing methods, such as pulsed-field gel electrophoresis [9]. Several tools for in silico *Salmonella* serotype prediction have been developed to utilize the available NGS data and eliminate the necessity of separate serotyping assays [10,11,12]. One such tool is the *Salmonella* in silico typing resource (SISTR), which predicts *Salmonella* serotypes using draft genome sequencing data [13]. SISTR detects the genetic determinants of O and H antigens, compares them to an established WKL serotype database, and refines predictions using core genome multi-locus sequencing typing (cgMLST) and phylogenetic analysis.

The evaluation of the different approaches to *Salmonella* serotyping is important in order to determine the suitability of each assay for routine use in public health laboratories. The purpose of this study is to compare *Salmonella* serotyping using CTS and SISTR and determine the concordance between the two approaches.

## 2. Materials and Methods

### 2.1. Salmonella Strains and Check&Trace Salmonella™ Molecular Serotyping

A total of 1397 clinical *Salmonella enterica* subsp. *enterica* and non-*enterica* isolates from Alberta, Canada, submitted to ProvLab from frontline laboratory sites between January 2017 and July 2018, were included in this study. Each isolate represented a unique case of human *Salmonella* infection in the province. *Salmonella* isolates were cultured onto a trypticase soy broth agar (TSBA; ProvLab, Edmonton, AB, Canada) and a single colony was selected to perform the Check&Trace *Salmonella* assay as per manufacturer’s instruction (CheckPoints Rapid Molecular Detection Software v4.9.0.2/2.1.0.19/26-1-2017) and detailed in the publication by Ferrato et al. (2017) [3]. This assay uses a DNA hybridization array system of different known markers, and the software automatically associates known patterns to report a serotype. For reporting purposes, any isolate for which a serotype could not be fully determined by the software was referred to the Public Health Agency of Canada–National Microbiology Laboratory (PHAC-NML, Winnipeg, Manitoba, Canada) to confirm the serotype using conventional methodology, whereby antigenic formulae and serotype determination were based on the White–Kauffmann–Le Minor scheme.

### 2.2. DNA Extraction and Genome Sequencing

A single *Salmonella* colony was selected and grown overnight in LB-Lennox 0.5% NaCl broth for DNA extraction using the Qiagen DNeasy Kit (Qiagen, Valencia, CA, USA) or Epicentre MasterPure Complete DNA and RNA purification Kit (Lucigen, Middleton, WI, USA) as per the manufacturer’s instructions. Extracted DNA was quantified using the Qubit^®^ 3.0 Fluorometer (Thermo Fisher Scientific Inc., Mississauga, ON, Canada). Genome sequencing of isolates was performed at the PHAC-NML Core Genomics facility (Winnipeg, MB, Canada). Sample libraries were prepared using the Nextera XT DNA library preparation kit (Illumina, Inc., San Diego, CA, USA), and sequenced using the MiSeq Reagent Kit V3 (600 cycles) and the Illumina MiSeq platform. Data from isolates with a sequencing depth greater than 40× were included in this study.

### 2.3. In Silico Salmonella Serotyping Using SISTR

NGS was performed on all 1397 *Salmonella* isolates and the resulting raw sequence files were uploaded to the Integrated Rapid Infectious Disease Analysis (IRIDA) bioinformatics platform [14]. IRIDA consolidates several bioinformatics analysis pipelines used to process NGS data. In silico serotyping was performed using SISTR as part of the IRIDA platform. The SISTR algorithm is as previously described [13]. Briefly, SISTR assigns *Salmonella* serotypes using draft genome assemblies by applying a novel 330 loci cgMLST scheme in combination with the characterization of the genetic determinants of O- and H- antigens.

### 2.4. Data Analysis and Concordance Assessment

Serotype data assigned by CTS and SISTR platforms were compared and the concordance between the two methods was evaluated. An isolate was considered typed on CTS if there was a match with a serotype in the database. If only a pattern number was generated without associating with a known serotype, the isolate was considered non-typed. Isolates that had discordant results between CTS and SISTR, or that could not be typed by either platform, were conventionally serotyped as described above. Serotypes predicted in silico by SISTR were excluded from the concordance analysis if isolates were missing one or more genes encoding the targeted antigens or if isolates had less than 297 of the 330 loci included in the cgMLST schema. The statistical software SPSS Statistics (IBM) was used to assess the agreement between CTS and SISTR designation for the twenty most frequent *Salmonella* serotypes.

## 3. Results

Serotyping was performed using SISTR and CTS on all 1397 clinical *Salmonella* isolates, with confirmation using conventional serotyping as required. These isolates belonged to 87 unique serotypes (77 *enterica* subspecies; 10 non-*enterica* subspecies). A total of 1345 (96.2%) isolates were successfully serotyped by both CTS and SISTR (Table 1) with only two conflicting results, where a *Salmonella* (I) 4,[5],12:i:- was typed as *S.* Typhimurium by CTS and a *S*. Virchow was typed as *S*. Javiana by CTS. The distribution of isolates in the 65 different *Salmonella* serotypes represented is shown in Table 1. *S*. Enteritidis (*n* = 725; 51.9%) was the most commonly observed serotype followed by *S*. Typhimurium (*n* = 101; 7.2%), *S*. Heidelberg (*n* = 53; 3.8%), and *S*. Infantis (*n* = 47; 3.4%). For the 20 most frequent serotypes seen in Alberta throughout this time frame, which represent 87.8% of all isolates, the agreement between the CTS and SISTR methods was excellent, with κ = 0.983 (95% CI 0.973–0.993, *p* < 0.005).

In this study, 37 isolates could not be serotyped by CTS, 2 isolates could not be serotyped by SISTR, and 13 isolates failed to be serotyped by both platforms, so conventional methods were used to determine these serotype (Table 1). The 15 isolates that could not be serotyped using SISTR were distributed across 12 different serotypes. A total of 21 serotypes were identified from the 37 isolates typed by SISTR but not CTS, and all of these isolates were concordant with conventional typing results. Of the 13 isolates that could not be serotyped by both SISTR and CTS, *S*. *enterica* subspecies *enterica* (I) 4,[5],12:b:- (possible Paratyphi B var Java, monophasic d-tartrate positive) was the most common serotype (*n* = 4) identified using the conventional method. Furthermore, these four isolates shared the same CTS microarray pattern.

## 4. Discussion

Rapid and accurate serotyping is crucial for *Salmonella* public health surveillance and outbreak management. Serotyping using CTS and the in silico serotype prediction tool SISTR was evaluated, and the two approaches were compared. Of the isolates typed by both assays, a high degree of concordance in the serotype assignments (99.9%; Table 1) was observed across a wide range of different serotypes, as only two isolates were discordant between the two methods. A total of 964 (69.0%) isolates included in this study were identified as one of the top five most common *Salmonella* serotypes seen in Canada [15]: Enteritidis, Typhimurium, (I) 4,[5],12:i:-, Heidelberg, and Infantis. Of these isolates, 963 (99.9%) were concordantly identified by CTS and SISTR. The one discordant isolate was *S*. Enteritidis, and this could not be serotyped by SISTR because the *fli*C gene encoding the H1 antigen could not be detected. Individually, both CTS (1346/1397; 96.3%) and SISTR (1382/1397; 98.9%) were able to accurately serotype the vast majority of isolates, and identified many uncommon serotypes (Table 1). The success rate observed for SISTR in this study is higher than previously reported values (94.8%) [8].

In total, 52 (3.8%) of the 1397 isolates included in this study could not be serotyped using CTS (*n* = 37), SISTR (*n* = 2), or both (*n* = 13). Of these, 11 isolates were identified as non-*enterica* subspecies. CTS was unable to serotype any of these 11 isolates; however, SISTR was able to identify the serotypes for 8 (Table 1). Several of the remaining isolates that CTS and/or SISTR had difficulty serotyping belong to rare subsp. *enterica* serotypes.

The ability of CTS to identify rare serotypes will be improved as the CTS database becomes more populated. Many non-typed isolates of the same serotype achieved the same numerical microarray pattern number, and so progressive updates to the CTS database (or local experience) can allow users to serotype isolates that had no previous association to a serotype in the software. In this study, four *S*. Newport and three *S*. Haifa isolates fell into this category (Table 1). For the three *S*. Haifa isolates, CTS detected differences in marker presence, but the serotypes assigned by the current version of the CTS software do not reflect the presence of these markers. Future updates of the CTS database and software will eliminate this discrepancy. Laboratories using CTS can also internally validate specific microarray patterns in order to report serotypes beyond the software update they are using.

A similar issue is observed when using SISTR as the cgMLST schema, whereby analysis relies on phylogenetic comparison to a serotype reference database [13]. The extent that rare serotypes and genetic diversity are represented in the SISTR database affects the serotype predictive ability of the algorithm. In this study, 15 isolates could not be serotyped by SISTR. Of these, 14 failed because they did not meet the minimum number of loci required for cgMLST-based serotype prediction. When the minimum cgMLST threshold score was less stringent, 11 of these 14 isolates had serotypes in agreement with CTS and/or conventional serotyping. Genome assembly quality also has a significant impact on the cgMLST threshold scores, and consequently the ability of SISTR to accurately predict the *Salmonella* serotypes [13]. A limitation of SISTR that will likely persist is its inability to confidently serotype isolates with partial antigenic expression. Monophasic serotype variants of *S*. Typhimurium are problematic for SISTR [16], and one discordant falls into this category. These isolates will require the additional characterization of individual antigens using conventional methods. However, it is important to note that isolates that fail to express an antigen remain problematic, and this is an underlying issue associated with relying on serotyping alone to identify genetic relationships between isolates. Serotyping is also prone to identifying spurious relationships. For these reasons, the cgMLST-based clustering algorithm used by SISTR should be the gold standard for determining relatedness, and the serotype should only be used as a screening tool. Serotyping results are an early indicator for potential clusters, alerting epidemiologists of situations wherein public health action may be necessary. The results of cgMLST-based clustering provide confirmation of suspect cluster cases and prompt further outbreak management.

There were two *S*. Virchow by SISTR that were not typed by CTS and were confirmed as *S*. *enterica* subspecies *enterica* (I) 6,7:r:-, a monophasic variant of *S*. Virchow, by conventional typing. For these two isolates, there were differences between the serotypes predicted by SISTR using cgMLST (*S*. Virchow) and antigen data (*S*. *enterica* subspecies *enterica* (I) 6,7:r:-). Since the SISTR algorithm favors the cgMLST schema when there is a discrepancy, *S*. Virchow was assigned to the two isolates. However, the serotype designation from the algorithm used by SISTR to infer antigen genes based on serogroup determination matched the antigenic formula achieved by conventional methods. This highlights the possibility of differences in serotype designation arising from shifts in the serotyping paradigm between different methods. For these reasons, traditional serotyping will still be necessary to identify the serotypes for isolates that could not be typed with confidence in silico.

Both CTS and SISTR serotyping are effective alternatives to conventional serotyping methods, which require technical expertise and quality control for the many antisera. The cost of performing serotyping using the CTS system is approximately CAD 60 per isolate, and is suitable for smaller laboratories that may not have access to capital for NGS equipment and bioinformatics pipelines for analysis, or do not have enough specimen volume to justify the upfront cost of NGS. Further, the hands-on processing and analysis for SISTR is labor-intensive and requires trained expertise, with the entire process taking a minimum of 4–5 days to complete. CTS features automated analysis with a practical turn-around time of 24 h from the isolation of the organism to reporting the serotype result, and it requires less technical expertise to operate.

Overall, both methods were largely successful at serotyping the *Salmonella* isolates in this study, and can drastically decrease the time and cost per isolate by providing an alternative method to conventional serotyping. SISTR is particularly useful if NGS data are readily available; however, more time requirement and expertise are needed to perform the analysis, while CTS produces comparable results without the high initial costs in equipment and staffing associated with NGS. In laboratories that are set up for NGS, the sequencing data used by the SISTR pipeline can also be analyzed to explore genomes for cluster detection, antibiotic resistance and other virulence gene markers, including the discovery of new genes. Although SISTR is not an ideal technique for detecting outbreaks or clusters in a timely manner as compared to the CTS system, it does provide an excellent platform to collect meta data for understanding the nature of the isolates. Both methods drastically reduce the time and cost per sample for ongoing serotyping compared to conventional methods.

## Figures and Tables

**Table 1:** Results of *Salmonella* serotyping of 1397 *Salmonella* strains from human clinical cases using the Check&Trace *Salmonella*™ (CTS) DNA hybridization microarray and *Salmonella* in silico typing resource (SISTR) method, or by the conventional agglutination method.

*Salmonella Enterica* Strains	Number of Strains Detected by:			
	CTS and SISTR	SISTR	CTS	Conventional Agglutination
**Subsp. *Enterica***				
Enteritidis	724		1	
Typhimurium	101			
Heidelberg	53			
Infantis	47			
Typhi	39			
(I) 4,[5],12:i:-	38 ^a^			
Newport	36	4		
Braenderup	26			
Javiana	25			
Agona	22			
Reading	20			
Paratyphi B var. Java	17	4		
Saintpaul	15			
Stanley	13			
Muenchen	12			
Sandiego	10			
Montevideo	6			1
Oranienburg	4		1	
Poona	4	1		
Indiana	3	1		
Virchow	3 ^b^	2		
Adelaide	1	1		
Give	1	1		
Apapa	0	1		
Birkenhead	0	1		
Carmel	0	1		1
Daytona	0			1
Grumpensis	0	1		
Haifa	0	3		
Litchfield	0			1
Miami	0			1
Muenster	0	3		
Offa	0	2		
Stanleyville	0	1		
(I) 4,[5],12:b:-	0	2		4
(I) 42:g,z_51_:-	0			1
Other 42 serotypes ^c^	125			
subsp. *salamae*				
(II) 16:m,t:-	0			1
(II) 48:d:z_6_	0	1		
subsp. *arizonae*				
(IIIa) 48:z_4_,z_24_:-	0	2		
subsp. *diarizonae*				
(IIIa) 41:z_4_,z_23_:-	0	1		
(IIIb) 60:r:e,n,x,z_15_	0	2		
(IIIb) 60:r:z	0			1
(IIIb) 65:k:z	0	1		
(IIIb) O Rough:c:z	0			1
subsp. *houtenae*				
(IV) 43:z_4_,z_23_:-	0	1		
TOTAL number of strains	1345	37	2	13

^a^ CTS result was *S.* Typhimurium for one isolate. ^b^ CTS result was *S.* Javiana for one isolate. ^c^ Other *S*. *enterica* subsp. *enterica* isolates detected less than 10 times and showing the same serotype from both CTS and SISTR methods.
